# Full genotyping and FAM19A4/miR124-2 methylation analysis in high-risk human papillomavirus–positive samples from women over 30 years participating in cervical cancer screening in Örebro, Sweden

**DOI:** 10.1371/journal.pone.0274825

**Published:** 2022-09-22

**Authors:** Malin Kaliff, Gabriella Lillsunde Larsson, Gisela Helenius, Mats G. Karlsson, Lovisa Bergengren

**Affiliations:** 1 Faculty of Medicine and Health, Department of Laboratory Medicine, Örebro University, Örebro, Sweden; 2 School of Health Sciences, Örebro University, Örebro, Sweden; 3 Faculty of Medicine and Health, Department of Research and Development, Örebro University, Örebro, Sweden; 4 Faculty of Medicine and Health, Department of Women’s Health, Örebro University, Örebro, Sweden; University of Ferrara: Universita degli Studi di Ferrara, ITALY

## Abstract

Currently, cervical cancer prevention is undergoing comprehensive development regarding human papillomavirus (HPV) vaccination and cervical cancer screening. In Sweden and many other countries, high coverage vaccinated cohorts are entering screening within the next few years. This entails demands for baseline HPV genotype data across the screening age range for surveillance and a basis for screening program adjustment. In 2016, Örebro County, Sweden, changed to primary HPV screening using HPV mRNA testing followed by cytology triage. An alternative triage method to cytology could allow for a fully molecular screening algorithm and be implemented in a screening program where self-sampling is included. Hypermethylation analysis of the human genes FAM19A4/miR124-2 has been suggested as a promising triage method. HPV mRNA-positive screening samples (n = 529) were included and subjected to genotyping targeting a broad range of both low-risk and high-risk genotypes in addition to hypermethylation analysis of the two human genes FAM19A4/miR124-2. Data were connected to cytological and histological status and age. The most commonly detected genotypes were HPV31, 16, and 52. In addition, HPV18 was one of the most common genotypes in high-grade squamous intraepithelial lesions (HSILs) samples. In relation to available vaccines, 26% of the women with histological HSIL or cancer (≥HSIL) tested positive for only hrHPV included in the quadrivalent vaccine and 77% of the genotypes in the nonavalent vaccine. According to these figures, a relatively large proportion of the HSILs will probably remain, even after age cohorts vaccinated with the quadrivalent vaccine enter the screening program. Hypermethylation positivity was associated with increasing age, but no HPV-related independently predictive factors were found. Accordingly, age needs to be considered in development of future screening algorithms including triage with hypermethylation methodology.

## Introduction

HPV infections are the underlying cause of cervical cancer (CC) and its precursors [1]. More than 200 genotypes have been detected in humans [2], and most HPV infections will resolve spontaneously; however, a persistent infection with certain genotypes will increase the risk of carcinogenesis. Twelve high-risk (hr) genotypes have been classified as group 1 carcinogenic, and additional genotypes have been classified as group 2A, probable carcinogenic, and group 2B, possible carcinogenic [3].

Internationally, CC is a major health issue with over 500,000 new cases yearly, and in some regions CC is the leading cause of cancer related death in women, much due to lack of prevention and access to healthcare [4]. In Sweden, the strategy for CC prevention is mainly focused on vaccination and screening. The quadrivalent vaccine Gardasil 4 (Merck Sharp & Dohme, NJ, USA) has been offered since 2012 and the nonavalent Gardasil 9 since 2020, and the first vaccinated whole-year cohort are expected to enter the cervical cancer screening program in 2023. With a future perspective in mind, knowledge of the HPV genotypes in a currently unvaccinated and well-screened cohort would contribute valuable information about baseline HPV distribution.

In Sweden, all women are invited to participate in cervical cancer screening from the age of 23 years. In August 2016, new guidelines were implemented in Örebro County whereby women over 30 years of age receive primary HPV screening followed by triage with cytology [5]. The screening method Aptima HPV assay, which targets viral E6/E7 mRNA, is used, with the method targeting mRNA aiming to address active infections where viral oncogenes are expressed, driving carcinogenic development [6]. HPV as a primary screening test has favorably higher sensitivity compared to cytology [7], which allows for longer screening intervals than cytology [8,9]. However, since the specificity is low, a triage method is needed. However, cytology is a resource-intensive and subjective method of analysis, not optimal for a screening using self-sampling, which has been introduced on a larger scale. Other triage methods have been discussed, and DNA hypermethylation analysis in certain host cell genes has been suggested as a promising molecular triage method comparable to cytology [10,11]. Unlike cytology, it could be implemented in a program with self-collected sampling, allowing full molecular screening. The value of methylation and genotype distribution in a population with an implemented HPV screening program has been less investigated. The aim of this project was to evaluate what HPV genotypes were present together with the FAM19A4/miR124-2 methylation status in the 500 first hrHPV-positive samples from women over 30 years participating in cervical cancer screening after conversion to primary HPV testing in Örebro, Sweden.

## Material and methods

**Fig 1:**
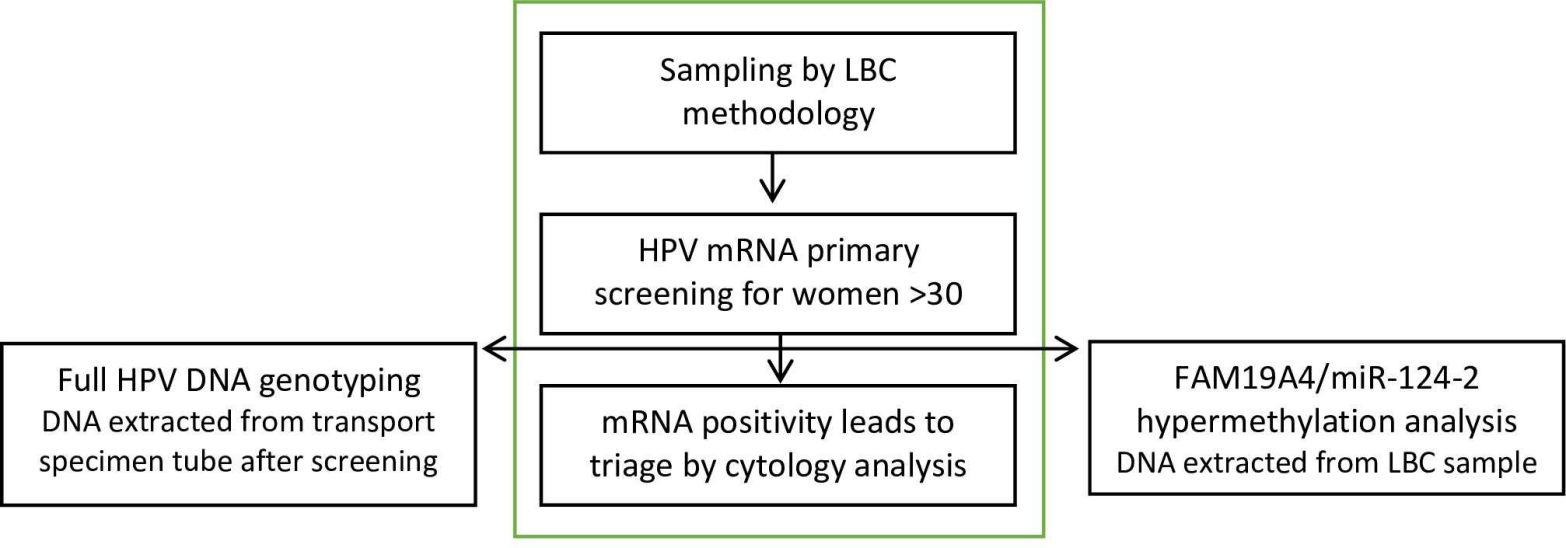
Study flowchart. The steps within the green middle box are part of the cervical cancer screening program in Örebro County, Sweden, where women 30 years and older are screened with primary HPV. All mRNA-positive results lead to reflex cytology analysis on the same sample with liquid-based cytology (LBC) method. For this study, all HPV mRNA–positive samples were subjected to full DNA genotyping (left) and methylation analysis of the host genes FAM19A4 and miR-124-2 (right).

Between October 2016 and April 2017, 7673 women over age 30 years (30–58 years) participated in cervical cancer screening in Örebro, Sweden. Within the screening program in Örebro County, professional sampling by liquid-based cytology (LBC) method (Presev Cyt, Hologic, MA, USA) is followed by primary HPV analysis with Aptima HPV assay (Hologic) detecting E6/E7 mRNA of the hrHPV genotypes HPV16, 18, 31, 33, 35, 39, 45, 51, 52, 56, 58, 59, 66, and 68 for women over age 30 years. Results are given as hrHPV positive or negative but without specific genotype information. Positive HPV mRNA screening results are followed by cytological triage according to Bethesda classification [12]. Women with normal cytology results are called for a new HPV test in three years, while deviant cytological results (≥ASCUS [atypical squamous cells of undetermined significance]) resulted in colposcopy referral. This, in accordance with national guidelines at the time of the study where histological classification were performed according to the World Health Organization Classification of Tumours of the Female Reproductive Organs, 2014 [13]. To reach 500 mRNA positive samples, samples from October 2016 to April 2017 were included in the study (n = 529) (Fig 1). Cases were defined as all those histologically confirmed ≥HSIL with a follow-up time up to three years (n = 70). The control group consisted of women with no evidence of disease, including women with normal, ASCUS, or low-grade squamous intraepithelial lesion (LSIL) in cytology, and normal or LSIL in histology (n = 447). Women with a non-assessable cytology sample or without follow-up were excluded (n = 12).

### DNA extraction

DNA from HPV mRNA–positive samples was extracted in two steps; first, directly from the Aptima transfer tube for DNA genotyping, and later from biobanked LBC samples for FAM19A4/miR124-2 analysis and for samples extracted from Aptima transfer tubes that were HPV negative.

Following the HPV screening analysis, 200 μl of liquid specimen from the Aptima transfer tube was used for DNA extraction in the QIA symphony system (QIAGEN, Hilden, Germany) using the QIAamp DNA Minikit and the Tissue LC 200 V7 DSP protocol with elution in 50 μl, according to the manufacturer’s protocol.

For 504/529 study participants, LBC samples from a biobank (Örebro biobank) were available for the second DNA extraction. A cell pellet from 100 μl of biobanked LBC samples was incubated with 180 μl ALT and 20 μl proteinase K at 56˚C on a thermoshaker for 2 h. In cell-abundant samples, extraction was performed with the QIA symphony system as described above. For samples with fewer cells, DNA was extracted in the QIA cube system (QIAGEN) with the QIAamp DNA FFPE Tissue Kit and tissue program with 50 μl elution volume according to protocol from the company.

### HPV genotyping

HPV genotyping on mRNA–positive samples was performed first on DNA extracted from specimen transport tubes. Second, analysis on all HPV DNA–negative samples was repeated with DNA extracted from LBC samples. Anyplex™ II HPV28 (Seegene, Seoul, Korea), which targets the viral L1 gene of 28 genotypes (HPV6, 11, 16, 18, 26, 31, 33, 35, 39, 40, 42, 43, 44, 45, 51, 52, 53, 54, 56, 58, 59, 61, 66, 68, 69, 70, 73, and 82) together with the human gene Beta-globin (HBB), was used for genotyping. The PCR was manually prepared and performed after manufacturer’s recommendations with approximately 50 ng DNA input into both multiplex reaction mixes per sample and was run on a CFX96TM Real-Time PCR System (Bio-Rad Laboratories, Hercules, CA, USA). The assay includes human internal control for every sample, genotype-specific plasmid controls for all genotypes, and non-template control in every run. Results were analyzed in the Seegene Viewer software (version 2.0). Melting curve analysis after 30, 40, and 50 cycles gives a semi-quantitative indication of the positive results.

### Analysis of promoter hypermethylation of FAM19A4 and hsa-miR124-2

Extracted DNA from LBC samples (≤200 ng) was subjected to bisulfite treatment using the EZ DNA Methylation-Gold Kit (Zymo Research, Irvine, CA, USA) according to protocol.

Approximately 50 ng of bisulfite-converted DNA was added into the methylation analysis. QIAsure (QIAGEN), a multiplex-methylation–specific rtPCR method, was used to evaluate the hypermethylation status of two human host cell genes (FAM19A4 and miR-124-2), including one internal control gene (ACTB). Cycle threshold values of the two targets in each sample were reported in relation to the internal control and a low copy number plasmid control, the calibrator. The assay was run on the Rotor-Gene Q MDx 5plex HRM (QIAGEN) system and results were automatically analyzed with the Rotor-Gene Assay Manager software.

In addition to an internal control validation for every sample, for each run a low copy number plasmid control, for example, the calibrator, and a non-template control, was included. Cycle threshold values of the two targets in the samples are reported in relation to the internal control and the calibrator. Detected hypermethylation in any of the two targets resulted in a positive test result.

### Statistics and ethical approval

Data calculations and table and figure preparation were carried out in Microsoft Excel 2010 (Microsoft, Redmond, WA, USA), and for statistical analyses IBM SPSS Statistics version 25 (IBM, Armonk, NY, USA) was used. Pearson Chi2 test was used for comparisons of proportions and binary logistic regression was used for analysis of predictive factors for hypermethylation (age, screening outcome and HPV genotype). In analyses where comparisons between proportions were made with stratification, Fisher’s exact test was used. For the statistical analysis, p < 0.05 was considered statistically significant. Due to rounding, percentages in text and tables may not always add up to 100%.

The study was approved by the regional ethical committee board in Uppsala, Sweden (D-nr. 2017/297). The ethics committee waived the need for consent, since data were pseudonymized for analysis.

## Results and discussion

### HPV genotyping

Of all 529 women who tested positive for HPV mRNA in the cervical cancer screening, 495 (94%) tested HPV DNA–positive in this study (hereinafter called only HPV positive). In 34 samples (6%), HPV DNA from the targeted 28 genotypes was not detected. In 59% (292/495) of the positive samples, a single HPV genotype was detected, and multiple genotypes, counting both hrHPV and lrHPV, were found in the remaining samples (203/495, 41%). Between two and seven genotypes were detected together (2 genotypes, 57%; 3 genotypes, 29%; 4 genotypes, 9%; 5 genotypes, 4%; 6 genotypes, 0.5%; and 7 genotypes, 0.5%). Regarding hrHPV, 87% (429/495) of the samples harbored HPV from the IARC1 hrHPV class. Most of these samples included one IARC1 genotype (348/430, 81%) and a minority (81/429, 19%) more than one (2 genotypes, n = 73; 3 genotypes, n = 7; 4 genotypes, n = 1). Out of 495 HPV-positive samples, 13% (n = 66) tested positive for solely non-IARC1 genotypes. They were predominantly from the IARC risk groups 2A and 2B, and most often HPV66 and or 68 (n = 44), which are also targeted by the Aptima mRNA assay. However, in 22 samples none of the genotypes targeted in the Aptima test could be found and were instead from the IARC2B group (HPV53, n = 2; HPV70, n = 12; HPV73, n = 1; and HPV82, n = 2), lrHPV, or unclassified (HPV6, n = 1; HPV44; n = 1; HPV54, n = 1; and HPV61, n = 2).

**Fig 2:**
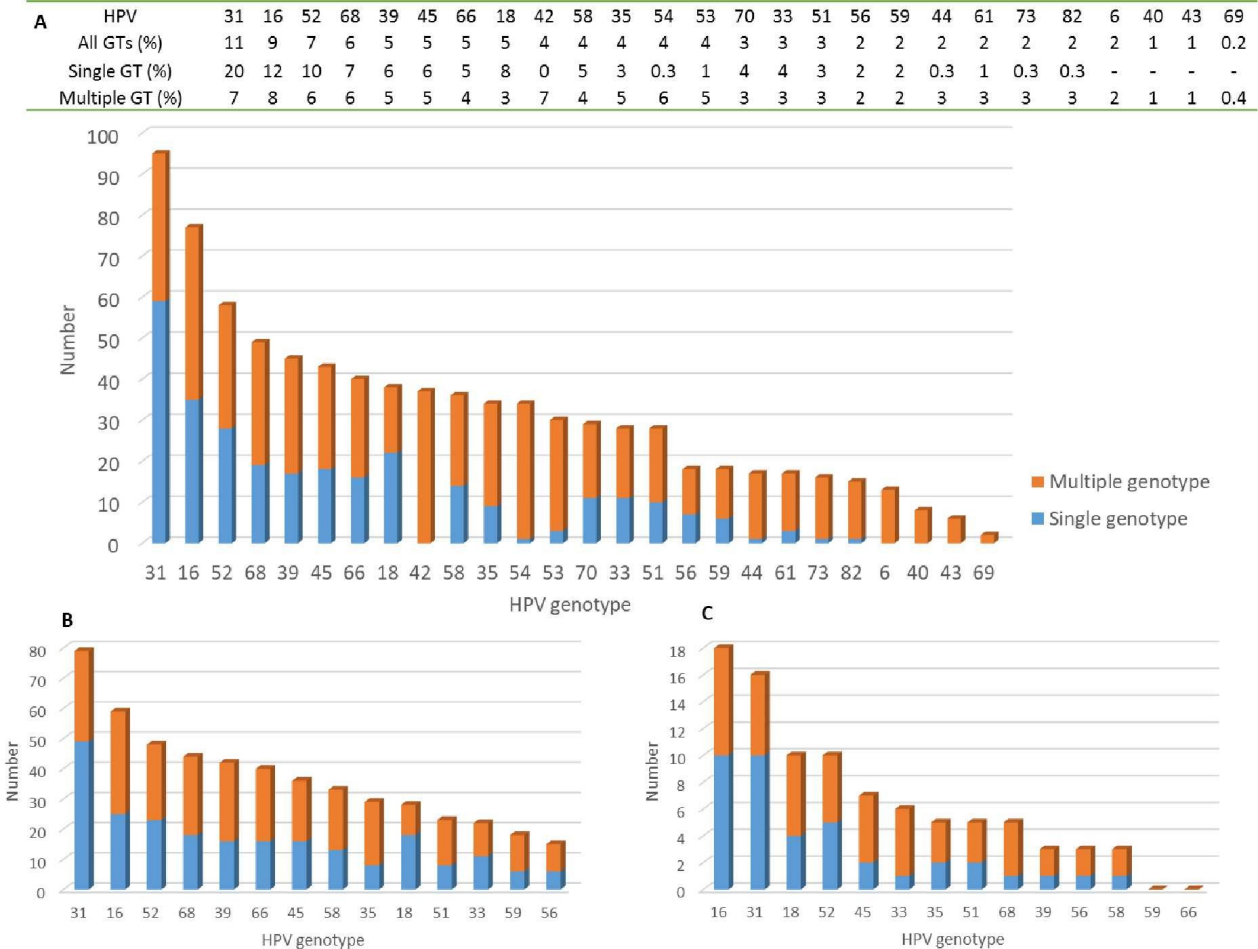
Total genotype distribution. Presentation of results from DNA genotyping from detected high-risk and low-risk HPV in both single- and multiple-genotype–positive samples in the screening population of women 30 years and older in Örebro, Sweden. **A.** All samples combined with numbers presented in the bar chart and proportions in table above. **B.** Samples among controls (normal cytology, ≤LSIL cytology, LSIL histology, and normal histology), including genotypes included in Aptima. **C.** Samples among cases (≥HSIL histology), including genotypes included in Aptima. HPV genotype is presented on the x-axis and number of positives for respective genotype on the y-axis. Blue bars constitute genotypes detected in samples with only one genotype (GT), and red bars represent GTs detected in samples containing more than one GT (multiple GT).

The most detected genotypes were HPV31 (11%), HPV16 (9%), HPV52 (7%), and HPV68 (6%). In single infected samples, HPV31, 16, and 52 were also most abundant, with HPV18 placing fourth, presenting the proportions of 20%, 12%, 10%, and 7%, respectively. The most common genotypes in multi-HPV samples were HPV16 followed by HPV31 and HPV42 (Fig 2A).

### HPV distribution in relation to screening outcome

The majority of the study group, 52% (n = 274), were aged 30–39 years, 33% (n = 172) were 40–49 years, and 16% (n = 83) were 50–58 years. In total, 517 out of 529 women had complete follow-up. Of these, 70 cases were defined as ≥HSIL (69 HSIL and one cervical cancer). In the control group with no evidence of disease (n = 447), there were 299 women with normal cytology, 61 with ≤LSIL cytology, 54 with LSIL histology, and 33 with normal histology. Of the 12 excluded women, six had a screening sample with non-assessable cytology result and six had no follow-up.

Regarding specific genotypes, as in the total group, HPV16 and 31 were the two most common genotypes (Fig 2B and 2C). HPV18 was the third most common in ≥HSIL samples compared to 12th most common in total and 8th in samples from women with no evidence of disease. HPV66 and 68, on the other hand, were less common in samples with confirmed histological ≥HSIL (Fig 2B and 2C). HPV DNA, as well as HPV16/18, specifically, was more commonly detected in samples with ≥HSIL compared to samples from women with no evidence of disease (p = 0.009 and p = 0.006, Table 1).

**Table 1:** HPV genotype distribution in groups of screening outcome. The HPV groups presented were positive vs. negative genotyping test, single vs. multiple genotypes within positive samples, positive for IARC1 genotype vs. positive for other non-IARC1 genotype, single vs. multiple IARC1 genotype within IARC1 positive samples, and HPV16/18 positive vs. positive for other IARC1 genotype. Results from statistical comparisons (Pearson Chi2) of proportions between the groups are presented in addition to strata analyses by age group in comparison of proportions between age groups (Fisher’s exact test). Total numbers of samples per category are presented in brackets by each category.

	Controls* (447)	Cases** (70)	Pearson Chi2	Fisher’s exact test by age strata
**HPV status**				
DNA pos (483)	413/447, 92%	70/70, 100%	p = 0.009 ***	30–39, p = 0.08
DNA neg (34)	34/447, 8%	0/70, 0%		40–49, p = 0.2
				50–58, p = 1.0
Single HPV genotype(284)	243/413, 59%	41/70, 59%	p = 1.0	30–39, p = 0.9
Multi HPV genotype (199)	170/413, 41%	29/70, 41%		40–49, p = 0.4
				50–58, p = 0.1
IARC1 pos (420)	352/413, 85%	68/70, 97%	p = 0.006	30–39, p = 0.03
Non IARC1 pos (63)	61/413, 15%	2/70, 3%		40–49, p = 0.2
				50–58, p = 1.0
IARC1 1 genotype (341)	288/352, 82%	53/68, 78%	p = 0.5	30–39, p = 0.5
IARC1 multiple genotypes (79)	64/352 18%	15/68, 22%		40–49, p = 1.0
				50–58, p = 1.0
HPV16/18 pos (110)	83/353, 24%	27/68, 40%	p = 0.006	30–39, p = 0.09
Other non-HPV16/18 IARC1 (310)	269/353, 77%	41/68, 60%		40–49, p = 0.05
				50–58, p = 0.4

*Controls (normal cytology, ≤LSIL cytology, LSIL histology, and normal histology).

**Cases (≥HSIL histology).

***Analyzed with Fisher’s exact test due to expected counts below five.

In the present cohort, the probability of having a histologically confirmed abnormality of ≥HSIL decreased with age (p = 0.006, Pearson Chi2 test). Also, women in the younger age group had a higher proportion of HPV IARC1 compared to the other age groups (p = 0.002, Pearson Chi2 test). In analysis by screening outcome strata, this association was, however, only statistically significant in samples with no evidence of disease.

All confirmed HSIL cases were HPV DNA positive (Table 1). The proportion of IARC1-positive vs non-IARC1–positive samples differed between the screening outcome groups (p = 0.006, Fisher’s exact test, Table 1). Among ≥HSIL samples, 97% (68/70) of positive samples were IARC1. The two samples of non-IARC1 classification were HPV68 and HPV70 from the IARC 2A & B risk groups.

Most ≥HSIL samples were positive for genotypes covered by available HPV vaccines, and 83% (58/70) contained at least one hrHPV vaccine genotype (HPV16, 18, 31, 33, 45, 52, and 58). Fewer samples, 77% (54/70), were exclusively positive for vaccine genotypes. Concerning only HPV16/18, 39% (27/70) of ≥HSIL samples were positive for one or both, and 26% (18/70) contained solely HPV16 or 18.

### Hypermethylation of FAM19A4 and hsa-miR124-2 genes

LBC samples were available for hypermethylation analysis in 504 cases, and data could be obtained in 487 samples. Analytical results were positive for hypermethylation in one or both of the targets in 32% (158/487) and negative in 68% (329/487).

Of samples with a hypermethylation result, loss of follow-up was evident in 11 cases, five had a screening sample with non-assessable cytology result and six had no follow-up. Of the 476, 61 cases were defined as ≥HSIL (the previously described cancer case had no follow-up due to lack of sample material), and 415 as control group with no evidence of disease.

The proportion of methylation positivity increased by severity of screening outcome and presented with 67% in ≥HSIL compared to 28% in samples from women with no evidence of disease (p < 0.001, Pearson Chi2 test) (Table 2A). Among hypermethylation-positive cases, a substantial number of positive cases were also found in the group of women with no evidence of disease (115/156, 74%).

**Table 2:** A. Results from FAM19A4/miR-124-2 hypermethylation analysis. Presented in comparison between groups of differing age, screening outcome, and HPV status. The HPV groups presented were positive vs. negative genotyping test, single vs. multiple genotypes within positive samples, positive for IARC1 genotype vs. positive for other non-IARC1 genotype, single vs. multiple IARC1 genotype within IARC1 positive samples, and HPV16/18 positive vs. positive for other IARC1 genotype. **B. Multivariate analysis.** Binary logistic regression analysis of predictive factors for hypermethylation positivity in the FAM19A4 and hsa-miR124-2 genes.

	Hypermethylation pos	Pearson Chi2 test
**Age in years**		
30–39	61/251, 24%	p < 0.001
40–49	60/159, 38%	
50–58	37/77, 48%	
**Screening outcome**		
Controls*	115/415, 28%	p < 0.001
Cases**	41/61, 67%	
**HPV status**		
HPV DNA pos	148/455, 33%	p = 0.9
HPV DNA neg	10/32, 31%	
Single HPV genotype	76/258, 30%	p = 0.1
Multi HPV genotypes	72/197, 37%	
IARC1 pos	133/393, 34%	p = 0.1
Non-IARC1 pos	15/62, 24%	
Single HPV IARC1 genotype	101/314, 30%	p = 0.2
Multi HPV IARC1 genotypes	32/79, 37%	
16/18 pos	93/298, 31%	p = 0.05
Other IARC1 pos	40/95, 42%	

Factor	OR	95% CI	P value
Age groups (30–39,40–49,50–58 years)	2.3	1.7–3.0	p < 0.001
Screening outcome (controls* vs. cases**)	7.5	4.1–13.9	p < 0.001
HPV16/18 pos vs. other IARC1 genotype pos	1.1	0.7–1.8	p = 0.6

Controls and HPV16/18 positive samples were set as references and age groups were analyzed as non-categorical covariates in ascending order.

*Controls (normal cytology, ≤LSIL cytology, LSIL histology and normal histology).

** Cases (≥HSIL histology).

The probability of a positive methylation result increased with increasing age of the woman. In the age group 50–58 years, almost half of the samples were positive (48%). There was a statistically significant difference in hypermethylation-positive proportion between the three age groups (p < 0.001, Pearson Chi2 test, Table 2A). Non-HPV16/18–positive IARC1 samples were numerically more often hypermethylated compared to other IARC1 genotypes (p = 0.05, Pearson Chi2 test, Table 2A). Age and screening outcome, but not genotype (16/18 vs. other IARC1 positives), were significantly independent predictive factors for hypermethylation (Table 2B).

In the ≥HSIL sample group, hypermethylated samples were numerically more often positive for multiple genotypes (22/41, 54%) compared to hypermethylation-negative samples (6/20, 30%; Pearson Chi2, p = 0.08). Also in samples with no evidence of disease, the hypermethylated samples were numerically more often positive for multiple genotypes (51/115, 44%) compared to hypermethylation-negative samples (116/300, 39%; Pearson Chi2, p = 0.3).

**Fig 3:**
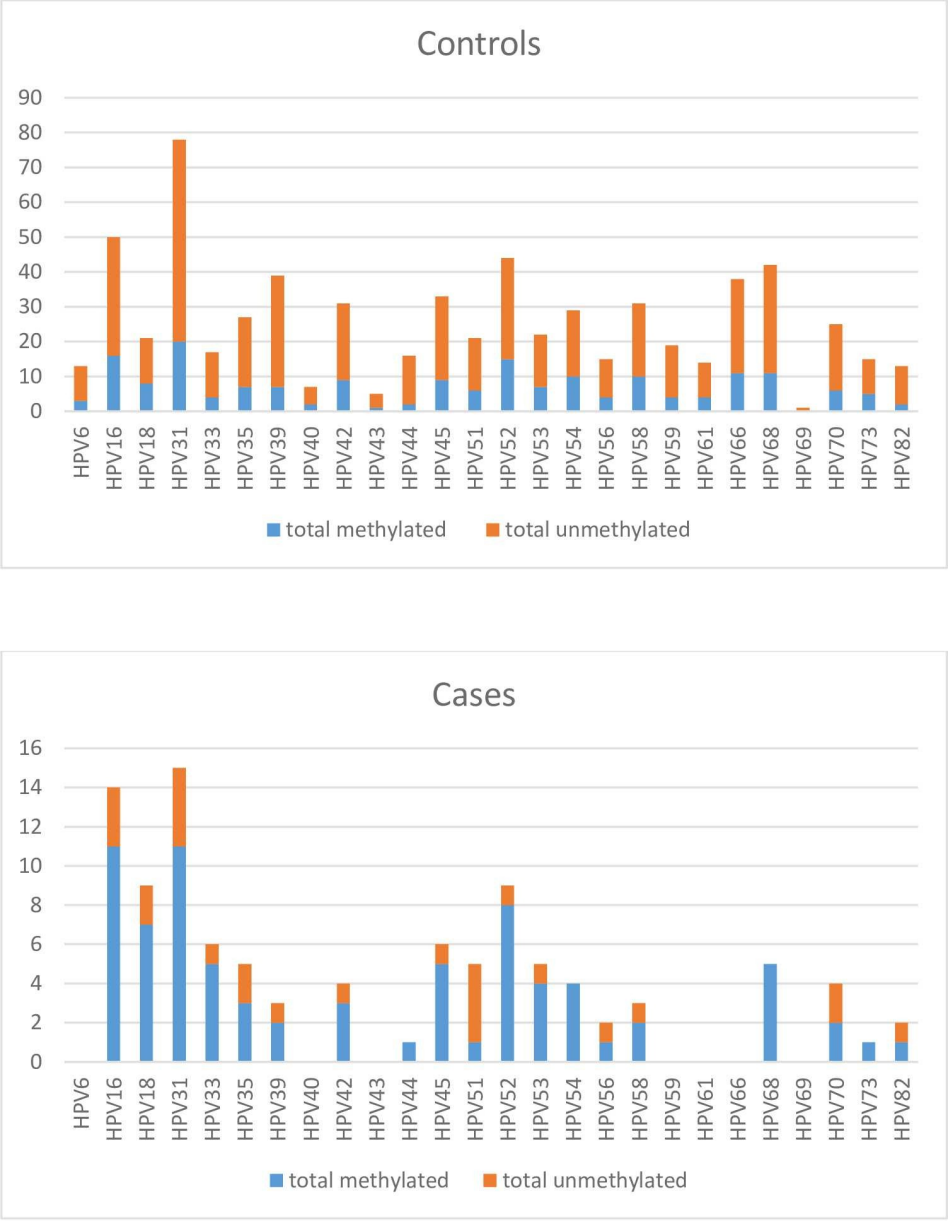
Genotype-specific hypermethylation patterns in samples among controls (normal cytology, ≤LSIL cytology, LSIL histology, and normal histology) and cases (≥HSIL histology).

Genotype-specific hypermethylation patterns in samples with no evidence of disease showed generally low frequencies of methylation in samples positive for all genotypes. In IARC1 genotype–positive samples, frequencies were between 18% and 38%. In ≥HSIL samples, the frequencies of methylation were generally higher, notwithstanding ≥HSIL samples positive for HPV51, where only 1/5 (20%) was hypermethylated and 4/5 (80%) unmethylated. In ≥HSIL IARC1–positive samples, the methylation frequencies were between 50% and 89%, with genotypes 52, 33, and 45 showing the highest methylation frequencies (Fig 3).

DNA negative samples (n = 34) presented invalid methylation results in two samples. The methylation rate in this group was 31% (10/32). In the group testing positive for non-Aptima targeting genotypes (n = 22), 18% of the samples tested hypermethylation positive.

## Discussion

The study cohort included women screened with primary HPV in the beginning of conversion to the new screening algorithm with primary HPV testing in Örebro, Sweden. Messenger RNA HPV–positive cervical screening samples were included in the study for genotyping, while previous studies have largely been based on cytological abnormalities. In this set-up, we are able to identify all active hrHPV infections in the screening population and provide information on the genotype distribution in the screening population.

The concordance in detection of HPV between the mRNA screening method and DNA genotyping was not complete, which is known from other studies [14,15]. A minority of the samples (10%) did not test positive for any of the genotypes targeted by Aptima; 6% tested totally HPV DNA negative. Cross-reactivity is one possible explanation previously described with similar numbers [16], and also described in the Aptima package insert [17], where HPV26, 67, 70, and 82 are stated to be targets of cross-reaction. HPV70 and 82 were represented in our samples but along with HPV6, 44, 53, 73, 54, and 61, suggesting additional cross-reaction, which has also been reported by others [18]. Proposed mechanisms for cross-reactions, beyond that of lrHPV genotypes, are specific or non-specific detection normally under cut-off limit, which in HPV multi-infected samples provides an added signal that pushes the signal over detection level, leading to a positive result [16].

The most common genotypes in the present study were HPV31, 16, and 52 both in total and in only single infected samples (Fig 2); in ≥HSIL samples, HPV16 was the most common. Similar data have been reported from other studies, including a Finnish study that also examined the prevalence of hrHPV in women who underwent cervical cancer screening, where the three most prevalent genotypes were HPV16, 31, and 52, and where HPV18 was the sixth most common [19]. In the present cohort, HPV18 was the eighth most common genotype in total, and the 12th among women with no evidence of disease compared with third most common in ≥HSIL samples, which is in line with a large Danish study [20]. The data reflect the potentially high-risk status of HPV18, which is the genotype, after HPV16 but similarly to HPV31 and 33, that poses the highest risk for developing CIN3+ [21]. HPV31 is apparent also in other studies [13,20,22] but not the most common as shown here, which is interesting, since HPV31 has shown to have the same risk as HPV16 for developing HSIL [23].

With implementation of vaccine programs, a considerable decrease in hrHPV in the screening population is expected. Data have already been reported on population level showing a decrease in HPV positivity and in precancerous lesions [24]. In the ≥HSIL group in the current study, almost all samples were IARC1 positive. Less than half (39%) were positive for HPV16 and/or 18, and the women would have been partly protected by vaccination targeting these genotypes, while 26% of the ≥HSIL samples were positive for only HPV16 and/or 18 and the women would have been fully covered. When estimating the same scenario with the nonavalent vaccine, 83% of the women with HSIL would be partly covered and 77% fully covered. However, according to these figures, a relatively large proportion of the cytological HSILs will possibly remain, even after age cohorts vaccinated with the quadrivalent vaccine enter screening [25,26]. It will be important in future screening programs to follow genotype occurrence in positive samples and to monitor to what extent these will possibly develop into HSIL or cancer, even though data from screened populations show that 80%–90% of cancer cases are positive with HPV types included in the nonavalent vaccine [25,26], as well as the higher risk of HPV16, 18, and 45 progressing to cancer compared to other genotypes [26].

Hypermethylation of the human genes FAM19A4 and miR124-2 has shown to detect cervical cancer with high sensitivity [11]. A negative methylation test reports risk comparable to that detected by normal cytology, for histological CIN3+ with up to 14-year follow-up [8]. Prospective studies on the test’s ability to predict regression or non-regression of a cervical abnormality are ongoing [27]. As hypermethylation of the human genes FAM19A4 and miR124-2 in studies has been associated with high-grade cervical dysplasia and cervical cancer, the test is interesting as a triage supplement to HPV genotyping in hrHPV-positive samples in the screening program [28]. Here, data on HPV genotypes were evaluated in relation to hypermethylation in the two human genes. The methylation rate was 28% in samples from women with no evidence of disease, with a considerably higher methylation proportion of 67% in ≥HSIL samples (Table 2A). However, 33% of the ≥HSIL samples were thus negative for hypermethylation in the target genes. Samples positive for IARC1 genotypes, except HPV51, were more methylated in ≥HSIL samples compared to control samples. Viral methylation itself has also been shown to be different between HPV genotypes. Comparing methylation in cases and controls, HPV51 had fewer hypermethylated sites compared to other high-risk genotypes [29]. Changes in methylation can lead to defective gene regulation and genomic instability, and for genes FAM19A4 and hsa-miR124-2, increased methylation can lead to gene silencing and thereby loss of tumor suppressor functions. Methylation of viral DNA has also been suggested to be a relevant biomarker for cervical disease, and combinations of viral and human targets are discussed [30]. Methylation is normally highly controlled, but HPV oncoproteins E6 and E7 interact with numerous epigenetic pathways, increasing methylation in both human and viral genes [31]. Hypomethylated HSIL samples, regardless of human or viral methylation, could hypothetically be examples of lesions that will regress spontaneously. For HPV51, studies have also shown this genotype to be the IARC1 genotype that most seldom develops into cervical cancer [32], speculatively due to less transforming and epigenetic changing abilities by its oncoproteins.

In addition, the results show that the hypermethylated samples were numerically more often positive for multiple genotypes. Multiple genotypes in one sample could potentially cumulatively increase the oncoprotein effects, thereby driving hypermethylation and transformation [31]. At the same time, former studies have shown that multiplicity is not an important factor for HSIL or cancer evolvement [25,33].

Women with a confirmed histological HSIL are most often treated due to the risk of progression to cancer in this group. Even so, it is known that HSIL is a heterogeneous group, where many lesions would spontaneously regress [34]. In this study it is not possible to determine whether a negative hypermethylation result can predict cytological lesions that are likely to regress. Even so, no HPV-related independently predictive factor for hypermethylation was found (Table 2A). This is reassuring for FAM19A4/miR124-2 hypermethylation for potential use as a triage method in a screening setting where there are expected changes of the genotype distribution ahead, as well as different choices of HPV assays.

In the present study, hypermethylation of the targeted genes was associated with age, where older women were more likely to present with a positive test result independent of screening outcome (Table 2B). Association of FAM19A4 hypermethylation and age has previously been evaluated by Luttmer and colleagues [35], demonstrating higher odds for a positive methylation result in women over 30 compared to women under 30 years of age.

This study presents the genotype distribution, with a broad range of targeted genotypes, both lr- and hrHPV and hypermethylation status of two human genes, in mRNA hrHPV–positive women participating in cervical cancer screening. The study population is a well-screened and essentially unvaccinated cohort, which makes it suitable as a baseline comparison in surveillance regarding future developments in screening populations. Future perspectives include linking genotyping and methylation data to data from upcoming screening rounds for these women. Further studies are also of importance to explore whether methylation and genotyping could be sufficient on self-collected samples.

## Conclusions

HPV genotyping in this study shows evidence that a relatively large proportion of histological ≥HSILs will remain, even after age cohorts vaccinated with the quadrivalent, as well as the nonavalent, vaccine enter screening. Except for age, no HPV-related independently predictive factors for hypermethylation were found. Accordingly, age needs to be considered in development of future screening algorithms, if including triage with hypermethylation and HPV genotyping.
